# Promising results following derotational femoral osteotomy in patellofemoral instability with increased femoral anteversion: A systematic review on current indications, outcomes and complication rate

**DOI:** 10.1002/jeo2.12032

**Published:** 2024-05-21

**Authors:** Antonio Klasan, Riccardo Compagnoni, Alberto Grassi, Jacques Menetrey

**Affiliations:** ^1^ AUVA UKH Steiermark Graz Austria; ^2^ Johannes Kepler University Linz Linz Austria; ^3^ 1° Clinica Ortopedica, ASST Centro Specialistico Ortopedico Traumatologico Gaetano Pini‐CTO Milan Italy; ^4^ Department of Biomedical, Surgical and Dental Sciences Università degli Studi di Milano Milan Italy; ^5^ IIa Clinica Ortopedica e Traumatologica, IRCCS Istituto Ortopedico Rizzoli Bologna Italy; ^6^ Swiss Olympic Medical Center, Hirslanden Clinique La Colline Geneva Switzerland; ^7^ Orthopaedic Surgery Service University Hospital of Geneva Geneva Switzerland

**Keywords:** distal femoral osteotomy, MPFL reconstruction, patella dislocation, patellofemoral instability

## Abstract

**Purpose:**

Patellofemoral joint instability (PFJI) can surgically be treated with a multitude of approaches, depending on the underlying pathology. In the presence of increased femoral anteversion, some authors have reported good results with a derotational distal femoral osteotomy (DeDFO). The purpose of the study was to investigate the indications, outcomes and complication rate of DeDFO for PFJI.

**Methods:**

A systematic review was performed according to the PRISMA guidelines (Preferred Reporting Items for Systematic Reviews and Meta‐analyses) by searching Medline, Embase, Web of Science and Cochrane Library databases through 1 December 2023. Included were levels 1–4 clinical studies of skeletally mature patients undergoing a DeDFO for PFJI irrespective of concomitant procedures. Study characteristics, indications, radiological and clinical outcomes, surgical technique and concomitant procedures, re‐dislocation and complication rate were all analysed, as was methodological quality.

**Results:**

A total of 12 studies including 310 patients (325 knees) were included. Three studies were cohort studies, all others were case series. The mean patient age across the studies was 22 years, and the mean follow‐up was 29.4 months. Femoral anteversion cut‐off was between 20° and 30°. Every study included at least one concurrent soft tissue, bony or combined procedure. Across all studies, one case of re‐dislocation was reported (0.3%) and four implant or osteotomy‐related complications (1.2%) were reported. All studies reported a statistically significant increase in clinical scores.

**Conclusion:**

This systematic review of DeDFO for patellofemoral instability in the presence of increased femoral anteversion demonstrates promising clinical results and an extremely low dislocation and complication rate. The heterogeneity of the cut‐off in anteversion and concomitant procedures, especially tibial tubercle osteotomy with seemingly identical results, indicates the need for high‐quality evidence for treating patellofemoral instability. Based upon this systematic review, we strongly recommend that DeDFO be added to the ‘menu à la carte’ of PFJI.

**Level of Evidence:**

Level III Systematic Review.

**Abbreviations**
DeDFO: derotational distal femoral osteotomy
MINORS: Methodological Index for Nonrandomized Studies
MPFL‐R: medial patellofemoral ligament reconstruction
PFJI: patellofemoral joint instability
PRISMA: Preferred Reporting Items for Systematic Reviews and Meta‐analyses
TTO: tibial tubercle osteotomy
TT‐PCL: tuberositas tibiae‐posterior cruciate ligament
TT‐TG: tuberositas tibiae‐trochlear groove

## INTRODUCTION

First‐time patella dislocation has an incidence of 23.2/100,000 person‐years [39]. The treatment recommendations in case of no associated injuries are fairly uniform in recommending conservative treatment first [9, 10, 23, 24, 26]. In case a re‐dislocation occurs, or the patient demonstrates apprehension [1] or reports pain [10], the issue at hand is more complex and is labelled patellofemoral joint instability (PFJI) [10].

Medial patellofemoral ligament reconstruction (MPFL‐R) has been the go‐to surgical treatment option [3, 30]. Outcomes of MPFL‐R as an isolated procedure are dependent on tunnel position [14, 34] and adequately identifying and addressing other underlying pathomorphological changes [10]. Historically, the four major anatomical factors leading to patellar dislocation are trochlear dysplasia, patella alta, excessive TT‐TG distance and patellar tilt [11]. The presence of all of these factors needs to be addressed in a systematic approach [10], since the re‐dislocation rate can be as high as 57%, especially in young patients, in patients with a history of contralateral dislocation and in the presence of trochlea dysplasia [51].

Excessive femoral anteversion, or antetorsion, has been a well‐established major risk factor for PFJI for decades [7], with or without the presence of valgus [52]. Due to the ‘internal rotation’ of the distal femur, the pulling force of the quadriceps is lateralized, causing a failure of isolated MPFL‐R [28, 29]. The proposed way to address excessive torsion is a derotational distal femoral osteotomy (DeDFO) [55].

Due to the complexity of the surgical procedure, reserved to high volume knee surgical centres, few recommendations are available on DeDFO in published algorithms and consensus papers [10, 23, 24].

Previous systematic reviews demonstrated the safety of the procedure with very low complication rates but also included DeDFO for patellofemoral pain [38]. A more recent systematic review demonstrated favourable results and a low redislocation rate but only included six studies [55]; however, both reviews included less than 200 patients. The purpose of this systematic review is to provide an update on the outcomes and complications of DeDFO in managing PFJI.

## MATERIALS AND METHODS

### Search strategy

A systematic review was performed following the PRISMA guidelines (Preferred Reporting Items for Systematic Reviews and Meta‐analyses) [31]. The Medline, Embase, Web of Science and Cochrane Library databases were searched by all of the authors independently on 1 December 2023. The search terms were as follows: (patellar dislocation OR patellar instability OR patellar subluxation OR patellofemoral dislocation OR patellofemoral dysfunction OR patella luxation) AND (rotational osteotomy OR derotational osteotomy OR de‐rotational osteotomy OR torsional osteotomy).

### Study selection

After the exclusion of duplicates using Zotero (Corporation for Digital Scholarship), all abstracts were screened by three authors (A.K., A.G. and R.C.). Any disagreements among the authors about a study's potential inclusion were resolved by the senior author (J.M.). All references of included studies were cross‐referenced with the included studies in order to ensure that no relevant articles were missing from the systematic review. Included were levels 1–4 studies investigating PFJI in skeletally mature patients where DeDFO was the primary procedure, leaving studies with concomitant procedures included. Minimum follow‐up was 12 months. Excluded were biomechanical, cadaveric and animal studies. Excluded were also surgical techniques, systematic reviews, non‐English language publications and duplicate cohorts.

### Data synthesis

Data extraction was performed independently by three reviewers (A.K., A.G. and R.C.). Each full‐text article was abstracted regarding study characteristics, patient characteristics, surgical techniques, outcome measures and complications. Any discrepancies were resolved through discussion with the senior author (J.M.). Outcome measurements were extracted as means and standard deviations. Study characteristics included publication date, study design, level of evidence, number of patients/knees and length of follow‐up. Surgical techniques for derotational femoral osteotomy and concomitant procedures were summarized according to the descriptions in the studies. Outcome measures consisted of pre‐ and postoperative clinical (patient‐reported outcome measures) and radiographic evaluations (degree of correction).

Complications included infection, delayed union, pseudoarthrosis, deep vein thrombosis, pulmonary embolism, vascular damage, nerve injury, compartment syndrome, fracture, stiffness or the need for reintervention.

### Risk‐of‐bias assessment

The methodological quality of each study was assessed independently by four review authors (A.K., A.G. and R.C.) according to the MINORS score (Methodological Index for Nonrandomized Studies) [42]. The items on the questionnaire were scored as follows: 0 if not reported, 1 when reported but inadequate and 2 when reported and adequate. The maximum possible score was 16 for noncomparative studies and 24 for comparative studies. MINORS scores of 13–16 for noncomparative studies and 21–23 for comparative studies were considered low risk of bias, and scores ≤12 and ≤20 were deemed high risk for noncomparative and comparative studies, respectively. Any discrepancies in scores were settled by consensus between the review authors.

### Data analysis

Meta‐analysis calculation was not possible due to study heterogeneity. Descriptive statistics were performed for all outcomes, with the data pooled for the re‐dislocation and complication rate. The degree of agreement for MINORS criteria was calculated using the Cohen *k* coefficient. In studies where standard deviation was not reported, it was calculated using the estimate described by Wan et al. [48].

## RESULTS

### Study inclusion and characteristics

**Figure 1:**
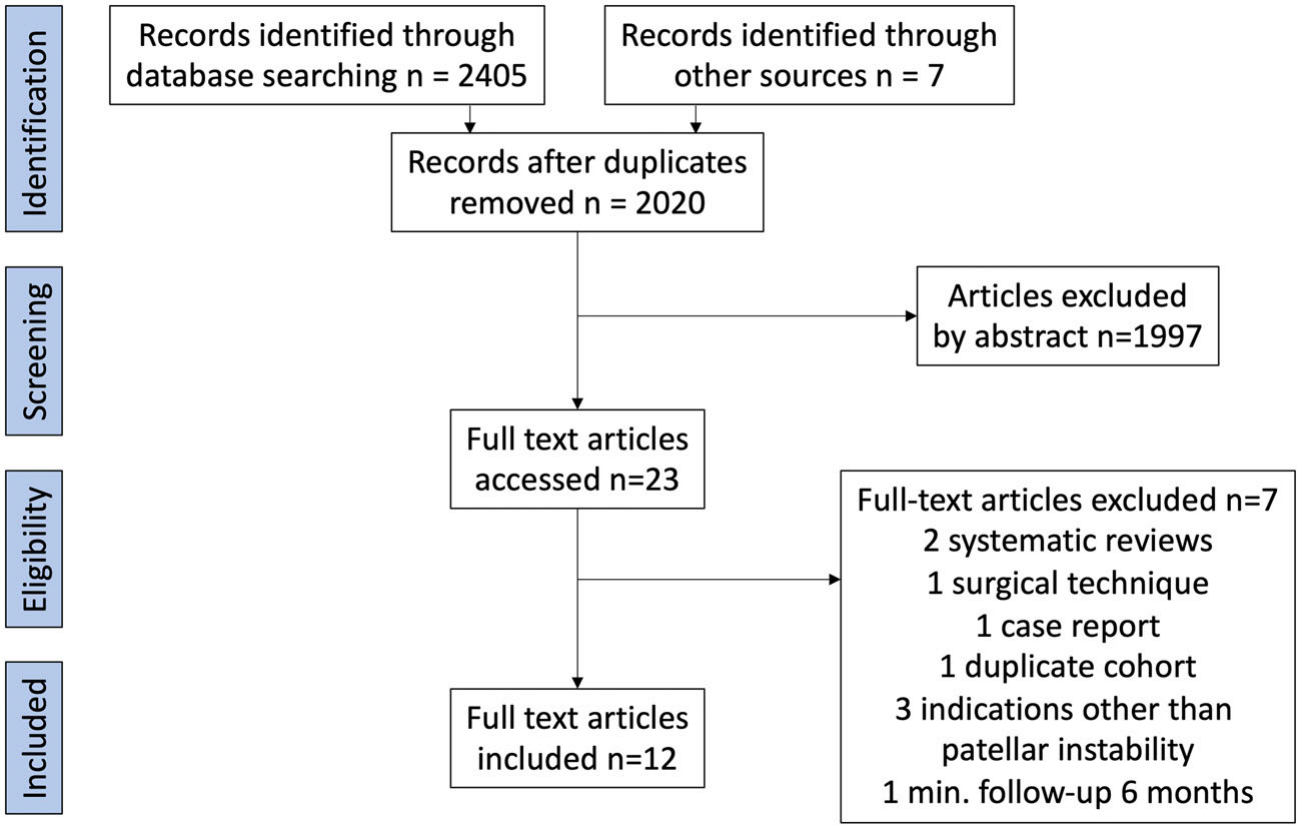
Flow chart of study inclusion.

After the application of inclusion and exclusion criteria (Figure 1), 12 studies were included in the systematic review. Four studies were retrospective cohorts [18, 20, 57, 60], and the rest were case series (Table 1). Cohen *k* was 0.9, indicating excellent agreement. One study was excluded due to an almost complete cohort overlap [59], and a partial cohort overlap of 5 years was observed with two studies [20, 25], which were left in the review. One study was excluded due to the inclusion of patients with patellofemoral pain [15].

**Table 1:** Study characteristics.

Author, year	Study design	MINORS score	Patients (knees)	Mean age [range]	Mean follow‐up, months
Biedert 2020 [4]	Case series	12/16	7 (9)	22	25
Cao 2022 [6]	Case series	13/16	14 (14)	19	30
Deng 2021 [12]	Case series	8/16	13 (13)	19	27
Frings 2018 [17]	Case Series	11/16	16 (19)	21	26
Hao 2023 [18]	Cohort study	21/24	31 (31)	24	36
Hinz 2022 [20]	Cohort study	11/24	27 (30)	24	38
Imhoff 2019 [25]	Case series	13/16	42 (44)	28	44
Nelitz [33]	Case series	12/16	12 (12)	18	16
Tian 2020 [45]	Case Series	12/16	16 (17)	21	27
Yang 2019 [54]	Case series	12/16	20 (20)	21	18
Zhang 2020 [57]	Cohort study	20/24	66 (66)	21	37
Zhou 2023 [60]	Cohort study	20/24	64 (64)	23	28

A total of 328 patients (339 knees) underwent a supracondylar DeDFO for patellofemoral instability. Mean patient age ranged between 18 and 28 years, and follow‐up ranged between 16 and 44 months (Table 1). Tibial tuberosity‐trochlear groove (TT‐TG) distance was not analysed postoperatively in one study [33], and six studies observed a significant reduction of TT‐TG, although no tibial tubercle osteotomy (TTO) has been performed [4, 12, 18, 45, 54, 60].

### Surgical data

Both medial and lateral approaches were utilized in the studies. The cut‐off anteversion was between 20° and 30°, while the amount of torsional correction ranged between 14° and 29°, with two studies not reporting the amount of correction [4, 33] (Table 2). Four studies utilized hip‐knee‐ankle magnetic resonance imaging (MRI) for assessment of anteversion [17, 20, 25, 33]; the rest used a hip‐knee‐ankle CT scan, using seven different measurement techniques (Table 2).

**Table 2:** Surgical data.

Author, year	Approach	Cut‐off value for osteotomy	Average amount of correction	Anteversion measurement technique	Concomitant procedure
Biedert 2020 [4]	Lateral	27°	Not reported	Murphy [32]	MPFL reefing, trochleoplasty
Cao 2022 [6]	Lateral	30°	29°	Takagi [44]	MPFL reconstruction
Deng 2021 [12]	Medial	25°	13°	Takagi [44]	MPFL reconstruction
Frings 2018 [17]	Medial and lateral	20°	28°	Waidelich [47]	MPFL reefing, TTO, valgus correction
Hao 2023 [18]	Lateral	25°	20°	Zhang [58]	MPFL reconstruction
Hinz 2022 [20]	Lateral	20°	14°	Schneider [40]	MPFL reconstruction, lateral release, trochleoplasty, double‐level osteotomy, facetectomy, TTO, vastus medialis transfer
Imhoff 2019 [25]	Lateral	25°	19°	Schneider [40]	MPFL reconstruction, TTO, valgus correction
Nelitz [33]	Lateral	25°	Not reported	Tomczak [46]	MPFL reconstruction
Tian 2020 [45]	Lateral^a^	25°	14°	Waidelich [47]	MPFL reconstruction, MPFL reefing
Yang 2019 [54]	Lateral	25°	15°	Franciozi [16]	MPFL reefing
Zhang 2020 [57]	Lateral	30°	24°	Zhang [58]	MPFL reconstruction, TTO
Zhou 2023 [60]	Lateral	25°	18° and 20°^b^	Not referenced	MPFL reconstruction

^a^ Lateral approach not explicitly mentioned, intraoperative figures demonstrate a lateral distal femoral plate.

^b^ Two degrees less correction was observed in the group without trochlear dysplasia.

In all studies, concomitant soft tissue, bony or combined procedures were performed. TTO was performed in five studies, either distalizing [15], medializing [17, 20] or both [25, 57], while one study excluded all TTO cases [6] (Table 2).

### Outcomes, re‐dislocation and complications

All studies reported significant improvements in clinical outcomes, regardless of the score utilized in the study (Table 3). A single case of re‐dislocation was reported across all studies, giving a re‐dislocation rate of 0.3% for this systematic review; moreover, most of the studies reported no complications, while the minor complications reported were wound infection, anterior knee pain needing MPFL release [17] or prolonged physiotherapy due to flexion deficit that resolved by the final follow‐up [18, 33, 45, 54], one case of screw loosening [17] and four cases of loss of correction/re‐osteoynthesis [20, 25]. Mal‐ or non‐union was not reported (Table 3).

**Table 3:** Outcomes and complications.

Author, year	IKDC score		Kujala score		Lysholm score		Re‐dislocation rate	Complications at final follow‐up
	Preop	Postop	Preop	Postop	Preop	Postop		
Biedert 2020 [4]			41.2	83.1			0%	None reported^a^
Cao 2022 [6]	42.9 ± 6.2	86.8 ± 6.0	51.0 ± 6.8	75.4 ± 5.1	49.2 ± 7.9	75.2 ± 7.2	0%	None reported
Deng 2021 [12]	51.4 ± 8.4	83.6 ± 7.27	57.5 ± 8.8	87.4 ± 4.3	59.9±	83.88 ± 6.5	0%	None reported
Frings 2018 [17]			47.7 ± 27	84.4 ± 16	40.5 ± 20.4	84.6 ± 15.2	0%	Wound infection (*n* = 1), anterior knee pain (*n* = 1), screw loosening (*n* = 1)
Hao 2023 [18]	53.8 ± 9.3	86.2 ± 10.0	54.5 ± 9.7	85.1 ± 7.7	56.6 ± 9.6	86.8 ± 8.2	0%	None reported^b^
Hinz 2022 [20]	54.6 ± 18.7	74.1 ± 15.0	55.6 ± 13.6	80.3 ± 16.7	58.6 ± 17.4	79.5 ± 16.6	*n* = 1 (3.33% knees)	Re‐osteosynthesis (*n* = 3).
Imhoff 2019 [25]	54 ± 13	65 ± 17			46 ± 21	71 ± 24	0%	Loss of correction (*n* = 1).
Nelitz [33]	60	85	69	92.5			0%	None reported^b^
Tian 2020 [45]	48.0 ± 11.2	72.6 ± 9.3	59.9 ± 7.9	80.7 ± 7.2	56.7 ± 10.5	77.9 ± 7.7	0%	None reported^b^
Yang 2019 [54]	70.6 ± 21.4	90.8 ± 14.3	72.4 ± 19.9	88.2 ± 12.3			0%	None reported^b^
Zhang 2020 [57]	56.7 ± 11.2	83.1 ± 10.4	53.8 ± 11.2	82.3 ± 8.4	58.2 ± 10.2	83.7 ± 9.0	0%	None reported
Zhou 2023 [60]^c^	53.2	75.3	51.6	74.1	53	73	0%	None reported

^a^ One case of persistent pain due to a cartilage defect was reported in the results, but not as a complication.

^b^ Flexion deficit and postoperative pain were reported, all resolving within a few months after surgery.

^c^ Averages of both groups were calculated since the authors reported no difference between the groups.

## DISCUSSION

The most important findings of the present systematic review are significantly improved clinical outcome, a low reported re‐dislocation rate and a low incidence of mechanical complications after a derotational DFO for patellofemoral instability, in combination with an MPFL reconstruction, at the very least. Another important finding is the significant reduction of TT‐TG distance in studies where no TTO was performed with re‐dislocation rate remaining 0%.

Patellofemoral instability is relatively common [39] and can be difficult to manage [22], and although treatment algorithms exist [10, 23, 24], the basic surgical treatment remains MPFL reconstruction due to its extremely low morbidity and excellent outcomes [3, 30]. In the present systematic review, most of the studies reported performing an MPFL in addition to the DeDFO. Biomechanical data for native knees is limited, partially due to the difficulty of pressure measurement of the patellofemoral joint [21]. Kaiser et al. demonstrated an increased lateralizing force vector in more than 10° femoral torsion [29]. The increased torsion occurs along the shaft and the distal femur [37]. The correspondent in arthroplasty is the internal rotation of the femoral component in total knee arthroplasty, which is considered a well‐described and known risk factor for alteration of patellofemoral joint mechanics, wear and stability [2].

Some of the most commonly cited algorithms for the treatment of patella instability do not include DeDFO as an effective surgical option [10, 50]. Similarly, a recent consensus did indeed recommend a rotational CT for assessing torsion [23], but any form of distal femoral osteotomy was not a part of the consensus questions [23, 24]. Is any additional procedure necessary? There is evidence that all other pathologies can be left untreated, and a systematic, isolated MPFL reconstruction can be performed with excellent results at 1 and 2 years [5, 13]. However, long term, one third of patients with isolated MPFL reconstruction have patellofemoral osteoarthritis [41].

A previous systematic review investigating complications after DeDFO concluded that the procedure is safe both for patellofemoral instability and for anterior knee pain [38], the former being observed in the present study as well. Another, more recent systematic review of six studies investigating outcomes and satisfaction found very similar results but concluded that there is no consensus on when to perform a DeDFO [55]. A very recent meta‐analysis of 11 studies found a redislocation rate of 1.1% and overall excellent results, including better results when compared to isolated MPFL [49]. In the present study, which included 12 studies, the minimum cut‐off was 20° of anteversion, but some studies reported it as high as 30° [6, 57]. In the present systematic review, seven measurement techniques were identified, with both MRI and CTs being used for evaluation. Kaiser et al. evaluated six measurement techniques of femoral torsion on cadavers [27]. The greatest difference in mean torsional value was observed between Waidelich et al. [47] and Hernandez et al. [19], 11°. If the cut‐off is set at 2°, this represents more than 50% of the value. More importantly, out of the six techniques evaluated by Kaiser et al. [27], only one was utilized in the studies included in the present systematic review, Waidelich et al. [47].

If concomitant procedures are evaluated, the indication for any procedure becomes more complex. The only osteotomy to be performed is a distalizing TTO in the case of patella alta or a medializing TTO in the case of an increased TT‐TG distance, or a combination of both [10]. In the present systematic review, TT‐TG was measured in studies where a medializing TTO was performed and was not performed, with seemingly matching results. TT‐TG distance serves somewhat as a proxy of increased femoral torsion since patients with increased antetorsion will inevitably have an increased TT‐TG [53]. Due to variation in rotation when taking the measurements, an alternative to TT‐TG has been suggested, TT‐PCL [43], although a recent consensus suggests TT‐TG to be superior for diagnosing patellar distal malalignment [23].

Patellofemoral joint pathology is complex in nature; in osteoarthritis patients, it has been labelled as the ‘forgotten joint’ due to its perceived clinical insignificance [8]. The present systematic review demonstrates excellent results with close to 0% re‐dislocation rate and a low complication rate for what is a seemingly complex procedure. The caveat is that the procedure is overall still rarely performed, which is apparent by the relatively low number of patients per study and the fact that the results come from experienced centres. This review answers some questions but asks significantly more. The cut‐off value for when the procedure should be performed remains between 20° and 30° and is largely dependent on the measurement technique. The amount of correction varies greatly. Comparatively, TTO, still a very complex procedure but arguably less complex than DeDFO, has been reported to have a significant risk of complications, 4.6%, with major complications at 3% [35]. The results of this systematic review suggest the opposite.

A recent study also found that patients with unaddressed femoral torsion >30°, where MPFL and a TTO were performed, have lower clinical outcomes than patients with less torsion [36]. In light of these findings, it might be advisable to systematically assess torsion in addition to TT‐TG and correct at the morphologically most altered bone [56].

Some limitations need to be noted. The studies included are of low level of evidence, demonstrate significant heterogeneity and are inherently biased due to a significant number of concomitant procedures that differ both in indication and in technique. The cut‐off for the procedure itself is also variable. Two studies had a slight overlap in the cohorts.

## CONCLUSION

This systematic review of DeDFO for patellofemoral instability in the presence of increased femoral anteversion demonstrates promising clinical results and an extremely low dislocation and complication rate. The heterogeneity of the cut‐off in anteversion and concomitant procedures, especially TTO with seemingly identical results, indicates the need for high‐quality evidence for treating patellofemoral instability.

## AUTHOR CONTRIBUTIONS

All four authors devised the study. Antonio Klasan, Alberto Grassi and Riccardo Compagnoni did the screening, data extraction and the review. Jacques Menetrey resolved discrepancies. Antonio Klasan wrote the first draft, and Alberto Grassi, Riccardo Compagnoni and Jacques Menetrey revised it. All authors have read and approved the manuscript.

## CONFLICT OF INTEREST STATEMENT

Antonio Klasan is an associate editor for the Journal of Knee Surgery and an Editorial Board Member of Archives of Orthopaedic and Trauma Surgery and Knee Surgery, Sports Traumatology, Arthroscopy. He has been paid for presentations by Arthrex, Implantcast and FH Ortho. Jacques Menetrey is an editorial board member of Knee Surgery, Sports Traumatology, Arthroscopy. All other authors have nothing to declare.

## ETHICS STATEMENT

Not applicable and the systematic review requires no consent.

## Data Availability

All studies included are openly available. Our analysis can be shared upon request.
